# Unlocking Success: Community Engagement for Enhanced HIV Care Outcomes

**DOI:** 10.21203/rs.3.rs-4999642/v1

**Published:** 2024-10-18

**Authors:** Sarah E. Wiehe, Tammie L. Nelson, Bridget Hawryluk, Unai Miguel Andres, Matthew C. Aalsma, Marc B. Rosenman, Michael S. Butler, Michelle Harris, Kem Moore, C. Dana Scott, Sami Gharbi, Lisa Parks, Dustin Lynch, Ross D. Silverman, J. Dennis Fortenberry

**Affiliations:** Division of Children’s Health Services Research, Department of Pediatrics, Indiana University School of Medicine; Division of Children’s Health Services Research, Department of Pediatrics, Indiana University School of Medicine; Research Jam, Indiana Clinical and Translation Sciences Institute; Division of Children’s Health Services Research, Department of Pediatrics, Indiana University School of Medicine; Division of Children’s Health Services Research, Department of Pediatrics, Indiana University School of Medicine; Ann & Robert H. Lurie Children’s Hospital of Chicago, Northwestern University Feinberg School of Medicine; Getting to Zero Community Engagement Panel; Getting to Zero Community Engagement Panel; Getting to Zero Community Engagement Panel; Getting to Zero Community Engagement Panel; Division of Children’s Health Services Research, Department of Pediatrics, Indiana University School of Medicine; Research Jam, Indiana Clinical and Translation Sciences Institute; Research Jam, Indiana Clinical and Translation Sciences Institute; Temple University College of Public Health; Division of Adolescent Medicine, Department of Pediatrics

**Keywords:** HIV, community engagement, stakeholder engagement, patient engagement, human-centered design, design thinking

## Abstract

**Background:**

Though social determinants are the primary drivers of health, few studies of people living with HIV (PLWH) focus on non-clinical correlates of insecure and/or fragmented connections with the care system. Our team has used linked clinical and multisector non-clinical data to study how residential mobility and connection to social services influence the HIV care continuum. We engage a diverse group of invested patients and community members to guide and inform this research. Our objective is to generate stakeholder-informed, research-based interventions that are relevant to the community, and to share our engagement approach and findings so that other researchers can do the same.

**Methods:**

Our research team partnered with the Indiana Clinical and Translational Sciences Institute’s Research Jam, to develop and implement a human-centered design research plan to engage with individuals with lived experience relevant to our research. We recruited a panel composed of PLWH as well as clinicians and individuals from agencies that provide medical and non-medical services to PLWH in Marion County, Indiana. We used a variety of human-centered design tools and activities to engage individuals during six sessions, with results informing our engagement and research activities.

**Results:**

Since the inception of the project, 48 individuals have joined the stakeholder panel. Thirty-five are actively engaged and have participated in one or more of the six sessions conducted to date. The panel helped guide and prioritize analyses, aided in identification of data missing from our ecosystem, helped interpret results, provided feedback on future interventions, and co-presented with us at a local health equity conference.

**Conclusions:**

We utilized community engagement to expand the scope of our research and found that the process provided value to both stakeholders and research team members. Human-centered design enhanced this partnership by keeping it person-centered, developing empathy and trust, increasing stakeholder retention, and empowering stakeholders to collaborate meaningfully with the research team. The use of these methods is essential to conducting relevant, impactful, and sustainable research. We anticipate that these methods will be important for academic and public health researchers wishing to engage with and integrate the ideas of community stakeholders.

## Plain English Summary

Many people living with HIV do not get the care they need to stay healthy. They may face many problems that make it hard for them to access or afford medical services. They may also have other problems, such as mental health or substance use disorders, unstable housing, or unreliable access to transportation. We want to understand how these factors influence the health of people living with HIV and find ways to help them overcome these barriers and improve their health.

We use information from many sources, including records from health and social services, to measure services received and health outcomes. We also work with a group of people living with HIV and/or who provide people living with HIV with support or care in our community. They help us understand what is important to them, what information we need, what the results mean, and what solutions we should try. So far, we have 48 people in this group. We have hosted six meetings where we shared and discussed our findings and asked for their input.

We think that involving people living with HIV and those who seek to serve them is critical to our research. These individuals with lived experience relevant to our research have given us valuable feedback and suggestions that we can use to help guide our research, making it more relevant, useful, and impactful. It can also benefit the people who participate, with stakeholders learning from each other and from us.

## BACKGROUND

Despite significant advances, human immunodeficiency virus (HIV) continues to be a major public health issue in the United States, with an estimated 1.2 million residents 13 and older living with HIV [1]. Discontinuous engagement in HIV care contributes to poor outcomes and, at least in part, to an estimated 32,000 new HIV infections annually [1, 2]. Despite decades of medical and public health programming meant to improve HIV outcomes and decrease disparities, one in four people living with HIV (PLWH) received no HIV care in 2021, and only one-third were virally suppressed [1]. These dismal statistics highlight the need for a different approach.

Most research into correlates of the HIV care continuum have focused on clinical data, leaving much to learn about non-clinical correlates among PLWH who have insecure and/or fragmented connections to the continuum of care. Many PLWH experience health inequities, which are differences in health status caused by social determinants of health (SDOH) [3]. SDOH are conditions into which one is born, grows, or lives (e.g., education, income, race) [3, 4], and they play a critical role in determining health outcomes by creating conditions that either support or hinder health [5]. Understanding and addressing the interaction between SDOH and the risk factors created by them is essential for improving health outcomes among PLWH. For example, research has shown that poverty, lack of access to education, stigma and discrimination, and limited access to high-quality healthcare significantly influence the risk of HIV infection, access to and engagement with HIV care, and the ability to achieve viral suppression [4, 6, 7]. Individuals evicted from their homes may lose their social support structure and access to medical care and then may have poor health outcomes. By incorporating SDOH risk factors and a consideration of the social services intended to mitigate them into HIV research, investigators can better understand the barriers to HIV prevention and care, develop more targeted interventions, and improve health outcomes for PLWH.

To enhance understanding of the impact of SDOH and social services utilization on HIV outcomes, it is essential to involve stakeholders in research. Engaging stakeholders, including PLWH, healthcare providers, agency representatives, and community leaders, ensures that research is grounded in the realities of those affected by HIV [8]. This approach enhances identification of relevant risk factors, the mitigation effects of social services, the development of interventions that are culturally and contextually appropriate, and the implementation of strategies that are more likely to be effective and sustainable. Stakeholder involvement can also facilitate dissemination and uptake of research findings by ensuring that interventions reach those in need and are integrated into practice and policy. By collaborating with stakeholders throughout the research process, researchers can gain insights into the complexities of risk factors for poor HIV health outcomes, co-design studies that address pressing needs, and contribute to the development of comprehensive strategies that improve HIV outcomes and promote health equity. Engaging stakeholders in HIV research is not only a methodological imperative but also an ethical one, ensuring that research contributes to meaningful change for individuals and communities directly affected by HIV. Stakeholder engagement is essential to conducting relevant, impactful, and sustainable research [9, 10], and is a cornerstone of *Ending the HIV Epidemic: A Plan for America* [11].

Human-centered design (HCD) is community-engaged research that is qualitative, person-centered, and collaborative. It offers a framework for developing solutions that are deeply rooted in understanding the needs, behaviors, values, and experiences of stakeholders. HCD maximizes stakeholder retention and generates interventions that are more relevant to the community [12, 13]; it also establishes a reciprocal relationship between researchers and stakeholders in which positions are well-articulated, needs are clarified, and everyone’s capabilities are utilized to co-create contextually-appropriate projects. Applying HCD principles to non-clinical HIV research allows for development of interventions and strategies that are not only scientifically sound but also resonate with the lived experiences of people affected by HIV. PLWH have historically been included in programs to reduce the burden of HIV (e.g., Ryan White Part A Planning Councils); however, most HCD research involving HIV focuses on clinical trials rather than on correlates of engagement in the continuum of care. For this reason, many non-clinical researchers lack information necessary to employ HCD for their projects.

We apply HCD methods to engage stakeholders for our project, entitled Getting to Zero. Stakeholders include PLWH, HIV clinicians, and individuals providing non-medical services to PLWH in Marion County, Indiana, an area selected for the *Ending the HIV Epidemic* project due to its HIV disease burden [11]. Our objectives are to: (1) better understand non-clinical correlates of insecure and/or fragmented connections with the HIV care system; (2) generate stakeholder-informed, research-based interventions that are more relevant to the community; and (3) share our engagement approach, tools, and findings so that other researchers can do the same. Our approach promises to yield unique insights from the community as we co-design solutions with them that are both innovative and deeply relevant, including interventions that are acceptable, non-stigmatizing, and appropriately weigh and address ethical and legal considerations that may be identified [14].

## METHODS

### Stakeholder Panel

We recruited stakeholders through individuals and organizations with which our team has strong, established partnerships, including infectious disease clinics, HIV/AIDS service organizations, and the Indianapolis Transitional Grant Area’s (TGA) Ryan White Part A Planning Council. These organizations provided prospective stakeholders basic information about our study and told them how to enroll if interested. The enrollment process was conducted via a secure survey, where prospective stakeholders provided demographics, their role (i.e., PLWH, clinician, or agency representative), and their contact information. Indiana University’s recruitment specialists then reached out to describe the panel’s purpose in more detail, including the expected time commitment (three-hour sessions, biannually, for up to five years), and answered questions. The risks and benefits of participation were discussed. Recruitment specialists emphasized potential stakeholders’ critical role as experts in the lived experience of a PLWH, a clinician, or an agency representative, and that their involvement would bridge that of participant and collaborator. Prospective stakeholders were sent a secure link from which they could read the informed consent and could agree to participate if they wanted to do so. They also had the opportunity to reach out to research team members to discuss additional questions or concerns they may have had prior to consenting and are able to withdraw at any time. We recruited a demographically diverse pool of 48 stakeholders: PLWH (N = 14), clinical providers serving PLWH (N = 11), and representatives of agencies serving PLWH (N = 23) in Marion County (Table 1).

The recruitment specialists review the panel annually to confirm each individual’s continued interest, as well as recruiting additional stakeholders to join. This increases stakeholder participation and enables us to maintain good representation of PLWH, clinical providers, and agency representatives throughout the study. Stakeholders are compensated $150 for completed activities, each of which takes about three hours. The study was reviewed and approved by the Indiana University Institutional Review Board.

### Engagement Team

Our research team has extensive experience working with patients and community stakeholders to address a variety of research barriers [12, 15–18]. In addition, our work with the stakeholder panel is guided by HIV experts. One is a clinician-scientist who has worked in HIV prevention and treatment since 1987. Another has expertise in population-level HIV data and epidemiology. She has also been affiliated with the Marion County Public Health Department’s (MCPHD) Ryan White HIV Services Program (RWHSP) and the Part A Planning Council for 12 years. These relationships facilitate trust-building and guide topics for panel activities. They contribute to an impactful and sustainable project that is relevant to the needs of the public health community and the PLWH that are served by it. Our team also includes an expert in bioethics and law. This allows us to work with the panel to explore the balance between implementing novel, health-maximizing interventions and carefully considering issues such as responsible data stewardship, communication, confidentiality, cultural competence, and safety, trust, reciprocity, stigma, and facilitation of care-seeking behavior.

Our research team partnered with the Indiana Clinical and Translational Science Institute’s Research Jam (RJ). RJ is a multi-disciplinary team of experts in HCD, health services research, communication, and visual design. RJ served as translators to bridge communication between researchers and stakeholders. RJ used an HCD approach to engage with stakeholders to learn their experiences, concerns, and ideas regarding the study. The engagements helped guide analyses throughout the study (i.e., to identify additional measures and interpret findings), as well as to anticipate and address potential issues with application of the study’s findings.

### Engagement Activities

Stakeholders attended virtual or in-person workshop-like sessions, known as Jams, to discuss the project, facilitate identification and exploration of concerns and challenges they face both in pursuit and receipt of services, and to develop, interpret, and disseminate community-informed proposals for alleviating such concerns and challenges. RJ used the HCD approach to put and keep stakeholders in the center of discussion, to develop empathy and trust with them, to empower them to externalize ideas and collaborate meaningfully, and to be comfortable with ambiguity in solutions during the development stage [19]. RJ framed agendas to reflect emerging and evolving findings from our research using an overarching question (e.g., How might we understand reasons for insecure care connections among PLWH? How might we mitigate risks associated with migration?). Each session began with a warmup activity, followed by one to four interactive, generative activities. These ranged from ‘explore’ activities that were directly in response to findings or ‘create’ activities in which stakeholders helped prototype solutions [20–22].

**Figure 1:**
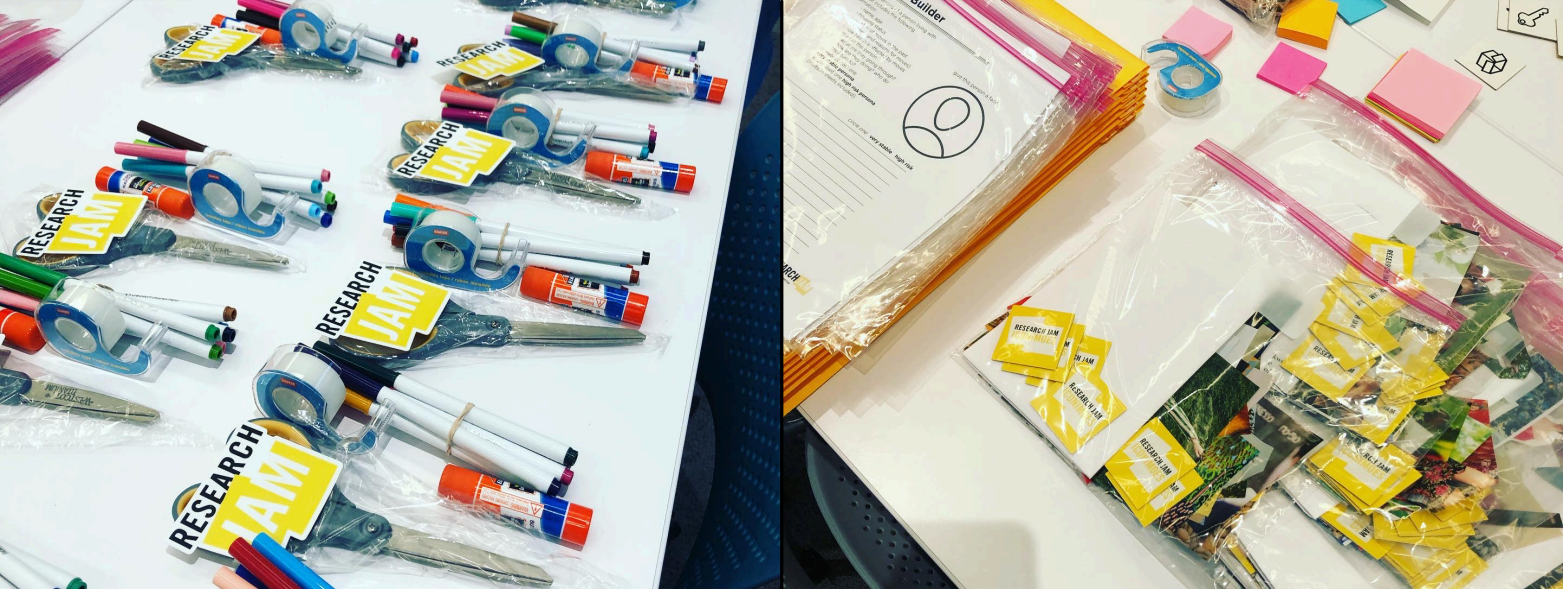


Originally, we planned to conduct biannual in-person Jams; however, the first Jam was in October of 2020, at the height of the COVID-19 pandemic. For this reason, we moved to a virtual setting, with the first four (Oct. 2020-May 2022) Jams conducted via group or individual Zoom calls. To recreate what would have happened in person, RJ mailed participants Jam kits with materials, worksheets, and supplies for use during each session (Fig. 1. Jam Supplies Mailed to Stakeholders in Advance). After activities were complete, participants shared their creations by video and emailed pictures to RJ. To date, we have engaged stakeholders in six Jams (Table 2), co-presented a panel discussion at a local health equity conference, and co-authored this manuscript.

We utilized several activities to successfully engage and, at one point, to re-engage stakeholders.

#### Hopes, Fears, and Ideals:

RJ began with an explore activity to become acquainted, build rapport, and investigate the needs and expectations of stakeholders. The HCD activity ‘Hopes and Fears’ [23] was modified to reflect ‘Hopes, Fears, and Ideals’. During this activity, participants were asked to verbally list their hopes, fears, and ideals regarding the panel, allowing us to reflect with them on ways to help it succeed.

#### Collage:

**Figure 2:**
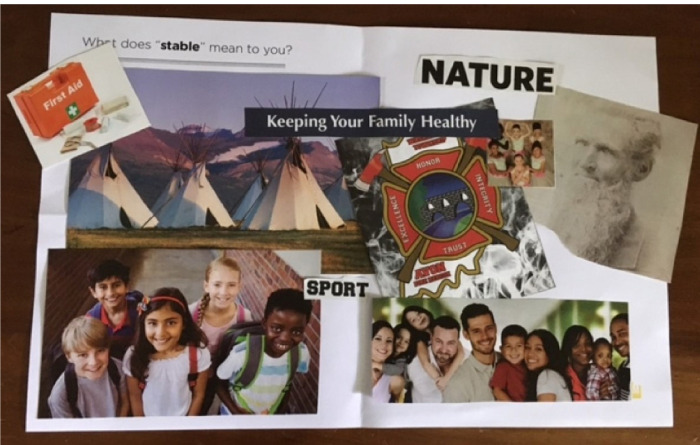


To explore stakeholders’ perspectives on risk and protective factors of a residential move, including harmful and advantageous neighborhood characteristics, RJ utilized three activities. The first was another explore activity called collage [24], during which participants glued magazine clippings to a piece of paper to answer a prompt (Figure 2. Jam 2 Collage Example). The prompt used during the Jam was: “What does ‘stable’ mean to you?” Stakeholders explained their collage to the larger group which led to in-depth discussion.

#### Personas:

**Figure 3:**
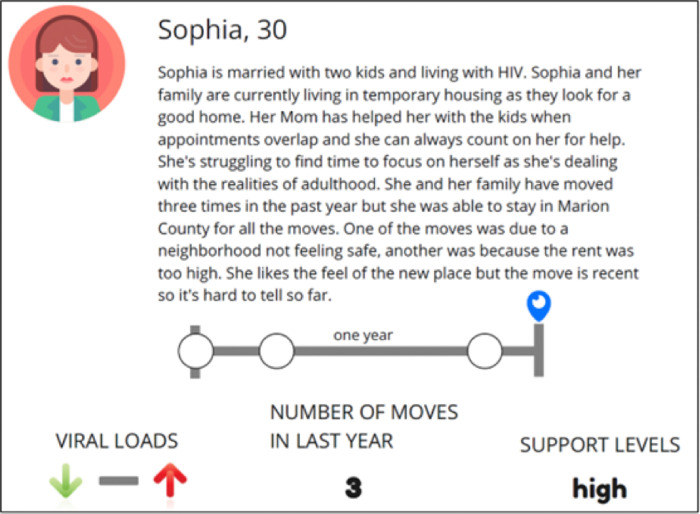


RJ used a create activity called Personas [25], during which they used fictional characters to depict traits and behaviors of the social group being designed for, and in this case, designed with (Figure 3. Jam 2 Persona Example), to get participants’ input on where the character could go for aid. RJ then asked participants to create personas to allow them to tell a story that reflected either a person who is “stable” or a person who is at “high risk,” in terms of receiving appropriate HIV care.

#### Mind Maps:

**Figure 4:**
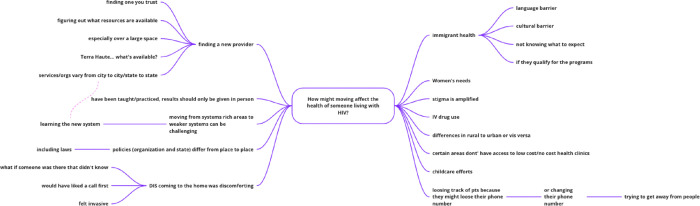


RJ utilized mind mapping, during which a diagram was designed around a central concept using associated ideas brought to life by earlier activities to build upon the ideas posed (Figure 4. Jam 2 Mind Map Example).

#### Red Flag Game:

RJ used a game called ‘Red Flag’ to identify whether stakeholders perceived an event as concerning in terms of health outcomes for PLWH and to identify events that might precede HIV-related health getting better or worse. RJ designed the ‘Red Flag’ game to explore when and for what reason(s) stakeholders would advocate for reaching out to a patient or client based on data sources available to the research team. Participants assigned red flags to those events they felt might lead to poor health outcomes. These items were placed onto virtual cards, and participants discussed the components of the data on each and were asked to decide whether they would reach out to the patient and why or why not.

#### Card Sorting Game:

RJ organized the Red Flag cards into frequencies, locations (e.g., rural area), and data events (e.g., arrest). Stakeholders then sorted the cards according to their perceived level of concern and discussed under what circumstances case managers should check in with a patient.

#### Decision Tree:

**Figure 5:**
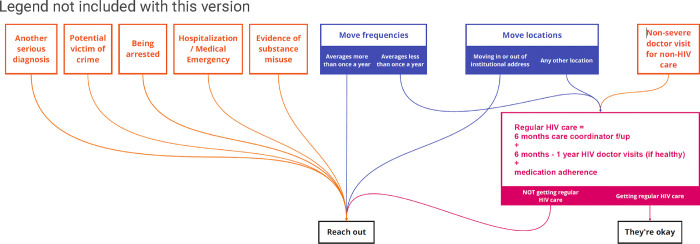


RJ worked with stakeholders to create a reaching out decision tree (Figure 5. Jam 3 Reaching Out Decision Tree) based on findings from the Red Flag and Card Sorting games.

#### Targeted Interviews:

Despite meeting recruitment goals, participation was limited to only 16 stakeholders through the first four Jams. For this reason, we interviewed a sample (N=12) of inactive stakeholders with the aim of understanding why so many had not attended and to hear ideas about improving engagement.

#### Information-Sharing Stations:

**Figure 6:**
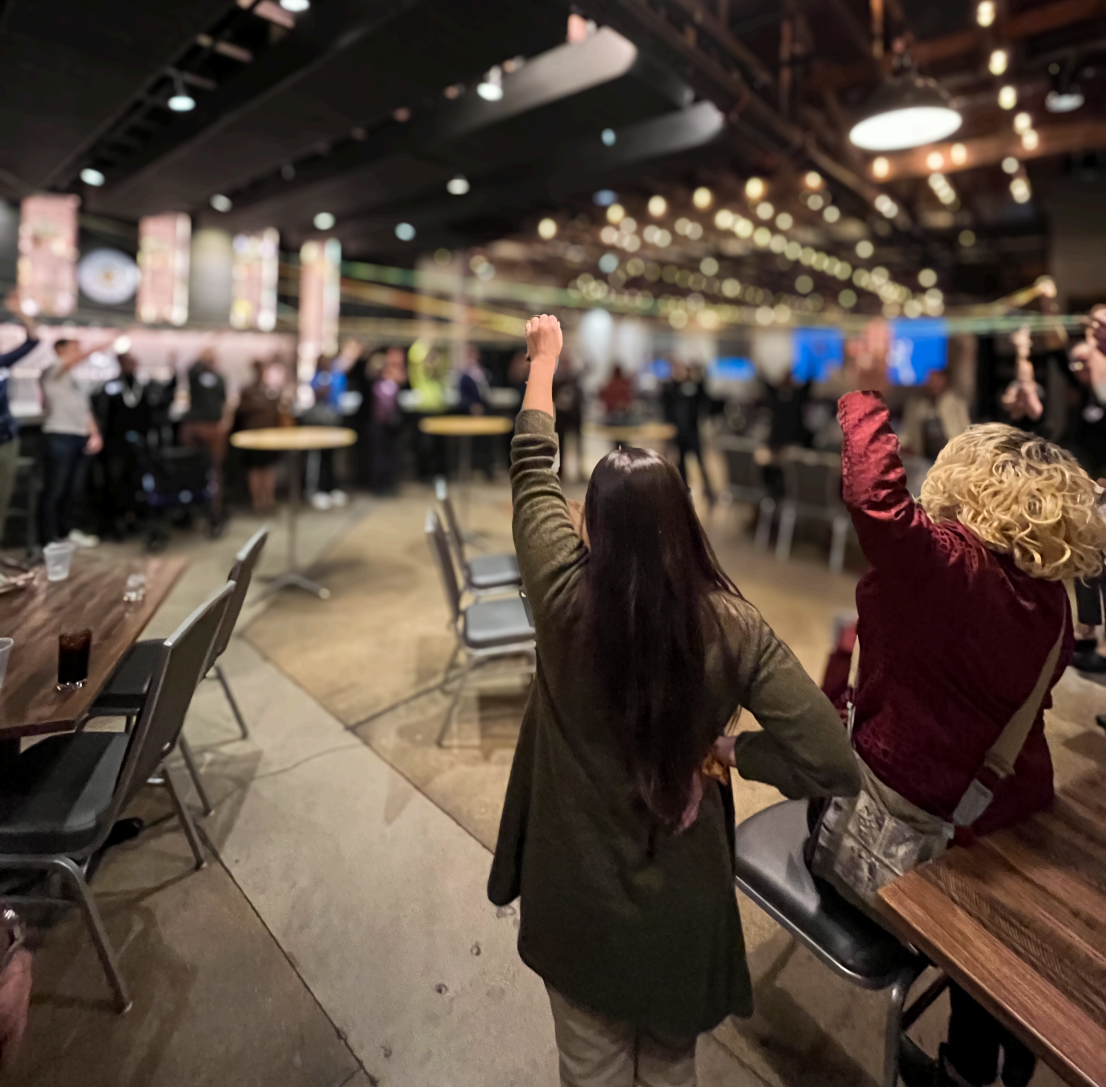


**Figure 7:**
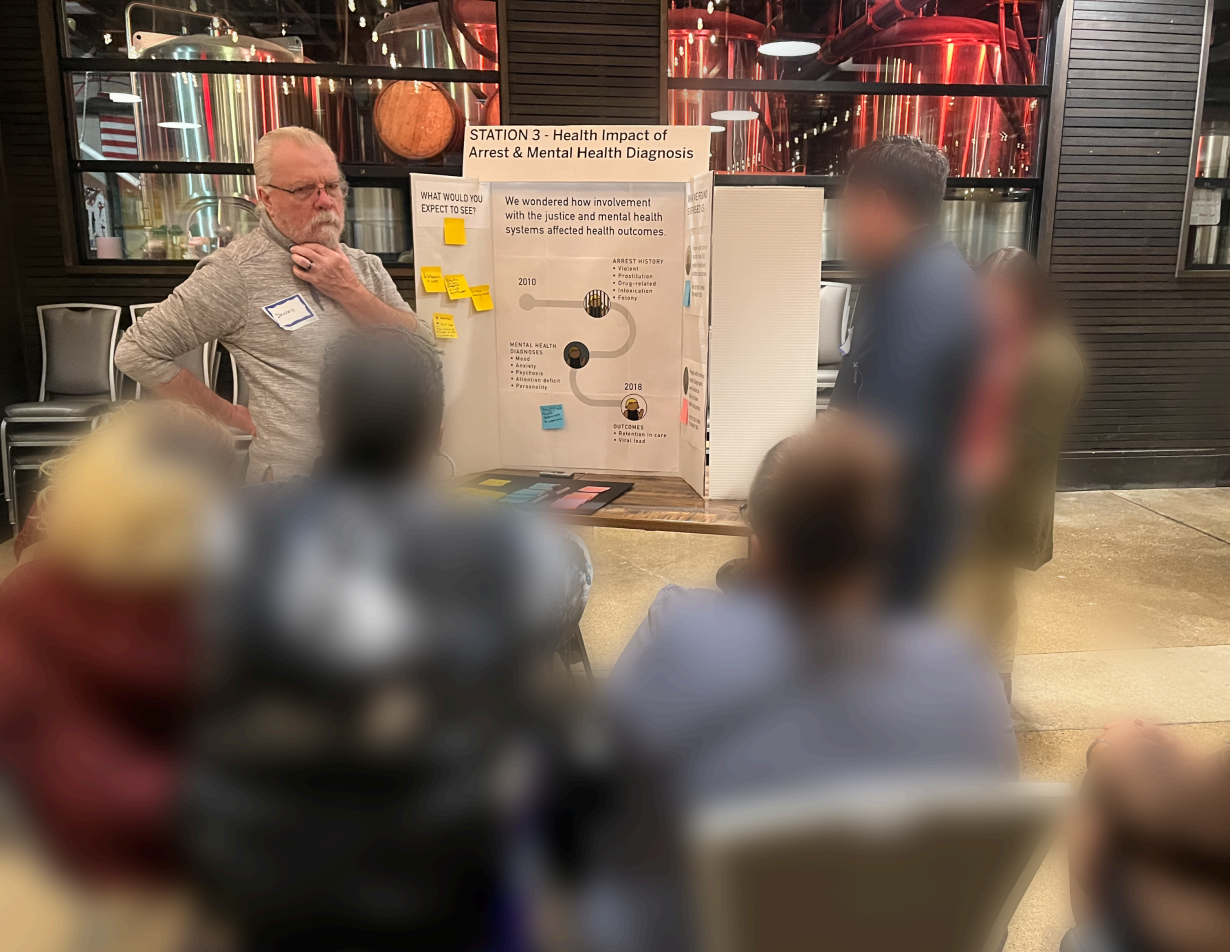


Guided by interview responses and then-current COVID-19 guidelines, we convened our next Jam in person. After a fun icebreaker (Figure 6. Jam 5 Icebreaker (Intentionally blurred to protect identities)), stakeholders rotated through five information-sharing stations, each hosted by research team members presenting a different topic (Figure 7. Research Team Member Sharing Information at a Jam 5 Sharing Station). This format offered stakeholders an opportunity to learn about our findings and to answer additional research team questions (i.e., What’s missing? How would you use this information?).

#### Brainstorming:

RJ facilitated a brainstorming activity, prompting stakeholders to list as many needs of PLWH as they could in the categories: medical, mental health, transportation, housing, social, financial, and other. Breakout groups then discussed resources available to address these needs and added ideas to bring resources together or to make them more accessible. They provided context by framing why some resources are more anticipated than others.

#### Resource Mapping:

**Figure 8:**
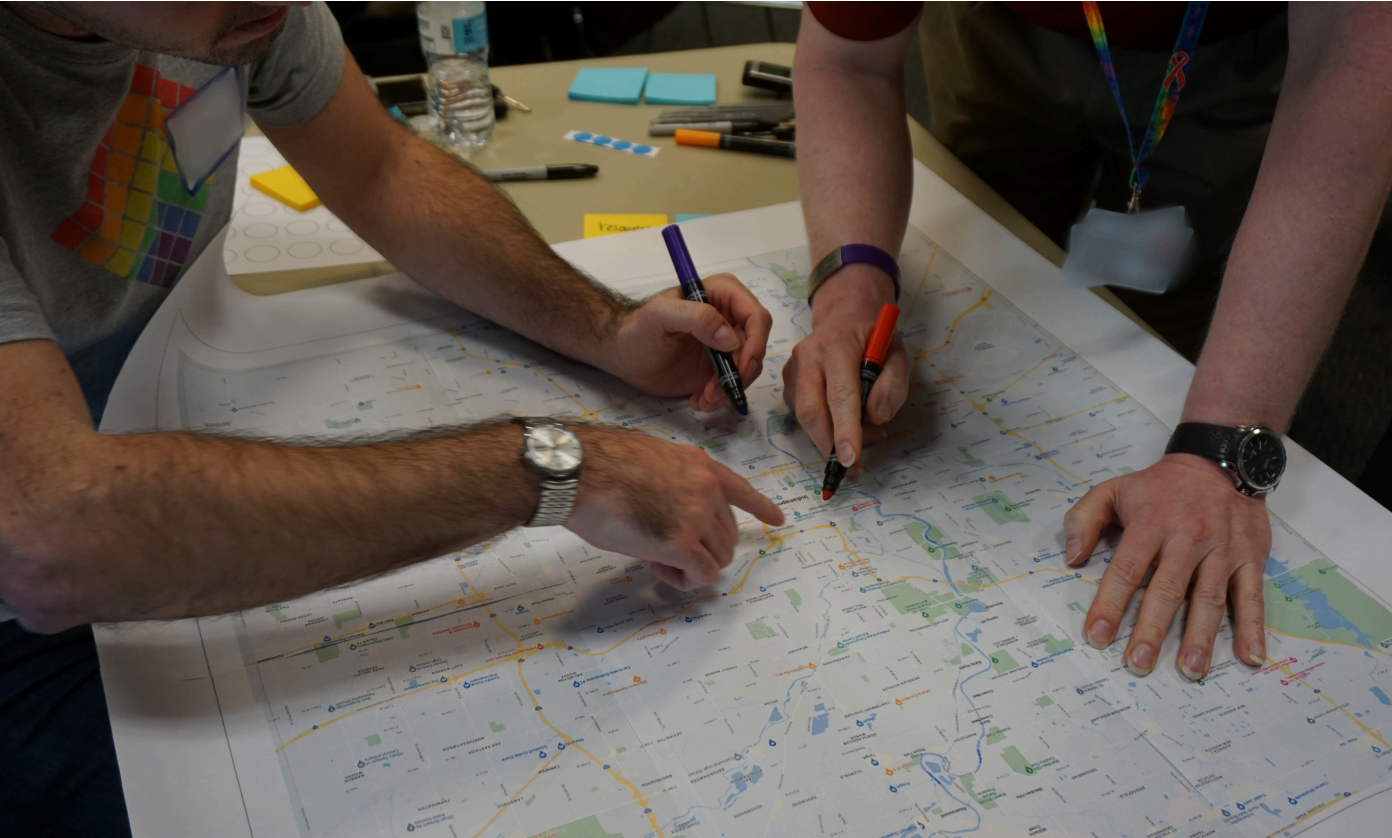


Following the brainstorming activity, stakeholders participated in an exercise in which they noted local resources directly onto county maps (Figure 8. Jam 6 Resource Mapping).

#### Microsoft Teams:

With the dual purpose of sharing session findings with those not in attendance and building upon previous sessions, RJ made session insights available to stakeholders online via Microsoft Teams, with opportunities for between-session discussion and discovery among all stakeholders. However, the stakeholder panel rarely uses this platform.

### Analysis

RJ assembled and analyzed Jam data using an online collaboration platform called Miro [26]. Data were separated into ‘snippets’ putting individual thoughts or concepts on separate virtual sticky notes for analysis and synthesis. Separating data into separate snippets allows for easy handling during the theming and analysis process [27]. Data were analyzed by 2–3 designers working collaboratively as they found themes and patterns with snippets. Ideas were externalized into affinity diagrams [23] and models to more easily discuss findings and to find consensus between designers. When building affinity diagrams, the designers were able to show patterns of, and the relationship between, data. This synthesis takes the designers beyond the data, as solutions and recommendations are explored and developed. RJ focuses on the abstract of ‘what could be’ as opposed to analysis, which focuses on the abstract of ‘what is’ [28]. RJ continued to use affinity diagrams and modeling during synthesis to develop solutions and recommendations before meeting with the research team to discuss how the information might be used to guide or complement their research. Synthesis was also performed by 2–3 designers. At times they worked individually then came together for discussion and collaborative development of proposed solutions.

## RESULTS

### Stakeholder Thoughts about Serving on the Panel

Stakeholders expressed hopes of increased collaboration between clinicians, agency representatives, and PLWH and felt that their participation in the project might lead to increased resources for PLWH (Table 3).

In fact, their participation did seem to lead to these benefits. Stakeholders later noted that Jams enabled easy information flow, providing everyone involved with a broad range of information. Multiple stakeholders asked to photograph Jam output/products and asked us to share meeting notes so that they could use the information for their own agency-related work.

Stakeholder fears centered on the impact that COVID-19 might have on meaningful collaboration, as well as other delays that might interfere with panel progress. Indeed, virtual versus in-person meetings did limit participation of many stakeholders. Lack of panel diversity was also a concern, as was a fear of poor panel outcomes (e.g., non-dissemination, barriers unchanged). Stakeholders offered ideas for working together for better collaboration and ‘outside the box’ thinking, with ideal outcomes to include reduced stigma, policy changes, and increased resources.

When interviewing inactive stakeholders, they expressed their desire to attend sessions but stated that their schedules make it difficult. They suggested more advanced notice of upcoming Jams with frequent reminders in a variety of formats (e.g., email, text, and mailers). Interviewees also expressed that in-person meetings and more clarity about compensation would increase motivation to attend. In response, RJ now sends early and frequent Jam reminders that include a clear statement of the compensation to be provided. Stakeholders’ preferred meeting locations were within proximity to downtown and to bus lines. As such, each of our in-person meetings met these criteria. Importantly, stakeholders expressed keen interest in learning about our findings to date, including how we responded to their prior feedback; and expressed an appreciation for the research team’s teachability, suggesting that we continue to listen, ask questions, and take their feedback seriously. This feedback, in addition to revised COVID-19 guidelines, led us to host our first in-person Jam, during which the research team shared findings back to stakeholders and solicited additional feedback.

Stakeholders conveyed concern that the panel was missing individuals newly HIV diagnosed; bias may result from having a subgroup of PLWH all of whom were diagnosed five-plus years ago. They suggested that recruiting these individuals might be difficult due to fear of participation (e.g., being ‘outed’), internal stigma, or being unable to participate due to excess time spent searching for resources. Concerns also included ‘same player’ burnout, because so many stakeholders participate in other HIV prevention and treatment efforts. These concerns led to discussion of methods our team can use to increase diversity of the stakeholder panel. Despite diversity of the panel in terms of race/ethnicity, gender, and member type, stakeholders reported concern that the panel does not represent the *cultural* mentality held by various PLWH. The mentality surrounding poverty was discussed in depth. For example, stakeholders offered that a PLWH in poverty and lacking resources may engage differently than someone who has never, at least not recently, experienced poverty. Early (childhood) trauma was also suggested as a mechanism behind insecurity and a reluctance to request assistance or to trust others. Ethnic culture, including family and spirituality, were also discussed, as some cultures are less open to others about discussing physical and/or mental health or are more stigmatized towards PLWH.

### Findings Informing HIV Research

#### Mobility/Migration:

Stakeholders agreed that PLWH seek good services, even moving long distances to access them, but that they often need to live near bus lines to access care. A number of factors may push PLWH from their homes, however, including stigma or violence, financial struggles or eviction, an inability to safely navigate one’s home, or a change in relationship status. These and similar reasons for a move equated to ‘forced mobility,’ which was unanimously seen as a risk factor for poor engagement in HIV care. Stakeholders agreed that a move might also serve as a protective factor for engagement in care, and that it depends on the reason for moving, financial status, and how well the move was planned. For instance, when a move results in enhanced social support, better healthcare, or safer or more stable housing, a move was seen as a protective factor. Regardless of the reason for a move, stakeholders agreed that researching access to medical and support services when planning a move could prevent lapses in care, and that the amount of time one had lived with HIV was a crucial factor based on developed relationships with providers and knowledge of navigating the system of care. Information gleaned from stakeholders led us to expand some data sources, to explore the acquisition of others, and to more deeply examine the contexts contributing to mobility and migration, including reasons for forced mobility. Specifically, we expanded to statewide Medicaid data, leveraged access to Enhanced HIV/AIDS Reporting System (eHARS) data for the full 10-county Ryan White Part A TGA, are integrating newly acquired Indiana eviction data, and are exploring the use of unemployment data. The newly added data and prioritization of the contexts of a move led to a need to hire a new data analyst. When doing so, we prioritized experience in geographic data and research. We are also considering time elements for future mobility analyses, including time since diagnosis, time spent retained in care prior to the move (a proxy for system navigation experience), and date of last HIV health care (a proxy for last provider contact).

#### Neighborhood Characteristics and Spatial Relationships:

Stakeholders agree that crime, poor SDOH indicators (e.g., unemployment, evictions), and lack of access to nutritious food and healthcare were among the harmful characteristics of a neighborhood (Table 4).

Advantageous characteristics included access to medical care, social and spiritual support, public transportation, and more. The importance of in-person healthcare was discussed, with distance and poor public transportation cited as reasons that some PLWH fall out of care. The idea of moving to access such services re-emerged. Access to a good social support system was also important for the prevention of isolation. This was a particular concern for older PLWH who had a distinct experience with HIV, with concern that some even discontinue medications due to isolation and loneliness. We worked with stakeholders to identify the physical locations of resources necessary to PLWH throughout Marion County. The research team will also integrate neighborhood characteristics and resources into future studies of mobility, migration, healthcare utilization, and HIV outcomes.

#### Social Services:

Stakeholders reviewed preliminary findings of Medicaid enrollment continuity and health outcomes among PLWH. Stakeholders agreed that continuous Medicaid enrollment improves access to care but said this often requires assistance applying and/or recertifying for coverage. Several stakeholders relayed firsthand experience of coverage gaps caused by a missed application deadline due to illness and/or the convoluted and confusing process. Stakeholders encouraged us to consider people with “emergency services only” enrollment as separate from those with other types of enrollment because these individuals are effectively uninsured. This feedback informed the methods we used in recent research on the impact of Medicaid enrollment discontinuity on HIV outcomes [29]. Stakeholders encouraged us to consider social services not already utilized by our team. Access to Ryan White services was prioritized, as were programs to assist PLWH who are unable to work. As a result, we are exploring options to access Ryan White CAREWare data to evaluate the interaction of Parts A/B/C coverage with other correlates of HIV health outcomes. We are also exploring unemployment data for inclusion in future analyses. Stakeholders identified housing assistance, food pantries, medical transportation, and help obtaining Americans with Disabilities Act-approved accessibility equipment as important to PLWH. For this reason, we are exploring sources of housing assistance data. Food pantries and medical transportation are fragmented in Central Indiana; however, we are considering CAREWare as a source of this data for those enrolled in the Ryan White Part A program that provides these services.

#### Behavioral Health Care and/or Incarceration:

Stakeholders were surprised to learn that an arrest and comorbid mental health diagnosis led to better health outcomes compared to PLWH without an arrest or mental health diagnosis [30]. Their surprise dissipated upon learning that these findings were explained by increased care utilization, likely from accessibility to various correctional system programs (e.g., Behavioral Court). Stakeholders suggested that incarceration alone can improve HIV outcomes due to increased access to structured care. Mental health and substance use disorder became key points of discussion. There was overwhelming agreement that behavioral health is highly impactful to HIV care outcomes, while also grossly underdiagnosed. Some PLWH do not seek out behavioral health services, while others seek care from private providers who may not communicate with other providers and/or feed data into larger systems (i.e., health information exchange). Stakeholder suggestions included improving care coordination between HIV and behavioral health care providers and agency workers. They also suggested evaluation of the timing of behavioral health diagnoses and/or incarceration in relation to HIV diagnosis or care outcomes of interest.

#### Local Resources:

Stakeholders identified needs and available resources in three broad areas. First, important sources of information to help PLWH find and research medical and behavioral health needs included libraries, Internet and social media, support people and groups, and churches. Second, physical resources important for PLWH were food pantries and delivery services, transportation and housing services, help obtaining Americans with Disabilities Act-approved accessibility equipment, education providers (e.g., academic institutions, back-to-work programs), and organizations providing second-hand items (e.g., clothing, household goods). Third, healthcare resources included medical transportation, help navigating medical needs, access to medical and substance use treatment providers, insurance navigation, telehealth, HIV/AIDS Service Organizations, and Ryan White services. Stakeholders indicated local resources meeting these needs directly onto maps (Figure 8). We plan to integrate these resources and other neighborhood characteristics into future studies to evaluate their impact on HIV care outcomes.

#### Case Manager Alerts:

We asked stakeholders to explore when, and for what reason(s), they would advocate for reaching out to a patient or client based on elements of data used in our research, including electronic health records, Medicaid claims, eHARS, incarceration, and address data. Whether an individual was receiving regular HIV care was the most important consideration; however, because not all HIV providers report to the state’s health information exchange, the use of electronic health records prevents comprehensive coverage of alerts. Public health use of eHARS was considered the most straightforward for alerts because it is a complete record of HIV labs and because MCPHD’s RWHSP already employs personnel who conduct outreach to PLWH who have fallen out of care. We learned from RWHSP stakeholders that outreach sometimes occurs months after an individual falls out of care, because of the retrospective method of reporting retention in care currently in use. We also learned that some PLWH reported for outreach are healthy individuals with undetectable viral loads who are required, by their physician, to receive only one viral load test per year. This leads to time wasted by outreach personnel. Because one of our research team members has extensive eHARS expertise and a trusted relationship with the RWHSP, and the RWHSP director is one of our active stakeholders, we were able to engage in an implementation project to leverage eHARS data to proactively generate alerts to identify PLWH who do not have a recent undetectable viral load and who are within 30–60 days of falling out care.

Stakeholders also identified additional data sources that could be useful in alerting case managers to situations of concern, including a mental health or substance use disorder (i.e., Indiana’s Data Assessment Registry for Mental Health & Addiction), HIV medication adherence (i.e., CAREWare), and data indicating a forced move (i.e., eviction data). Figure 5 illustrates the decision-making process stakeholders endorsed.

### Dissemination

In November 2023, we collaborated with stakeholders to develop and co-present our engagement methods at the Analysis to Action Health Equity Symposium [31]. We shared information about why we work with stakeholders, how we recruited them, our HCD approach, and how stakeholder input has enriched and changed our research. Three stakeholders attended and shared their experiences throughout the project. Attendees were interactive during the session, presenting the research team and stakeholders with questions. Attendees were most interested in stakeholders’ experience and in how we recruited and engaged nearly 50 stakeholders for a five-year project. This manuscript is an expansion of that presentation. Stakeholders worked with us to develop the content of, and to co-author, this manuscript, leading to robust discussion of the strengths and limitations of our work together.

## DISCUSSION

We utilize HCD and work with a diverse group of stakeholders impacted by HIV, as well as those who serve them, to better understand non-clinical correlates of insecure and/or fragmented connections with the care system and to generate stakeholder-informed, research-based interventions that are more relevant to the community. We are sharing our approach, tools, and findings so that others can do the same.

The stakeholder panel constitutes 48 individuals, 35 of whom remain actively engaged after nearly four years. Recruiting a large, diverse, and engaged panel for a five-year commitment required much effort, including building and leveraging trusted relationships with infectious disease clinics, HIV/AIDS Service Organizations, MCPHD, and the TGA’s Ryan White Part A Planning Council. Institutional Review Board approval and informed consent were necessary, as were considerations for privacy between and among stakeholders and researchers. Funding was necessary not only to pay study personnel and subject matter expert salaries and study expenses, but also to fairly compensate stakeholders. RJ engaged with stakeholders during virtual and in-person Jams and utilized a variety of HCD tools and activities to glean information for our study. RJ then assembled the data and conducted analyses to identify patterns and insights in the data before meeting with the research team to discuss how the information might guide or complement ongoing research. Exploring stakeholders’ hopes, fears, and ideals about participation was important for engagement. Their ‘outside the box’ thinking and fear that results might not be disseminated led us to collaborate outside of the planned Jams, leading to a collaborative conference presentation and co-authorship of this manuscript. When participation waned, we turned to inactive stakeholders to learn why. Their feedback led us to send earlier and more frequent reminders of Jam sessions, to offer more clarity on compensation, to host in-person meetings in a central location near a bus line, and to share findings with them. These changes were successful in increasing and maintaining stakeholder engagement.

The stakeholder panel helped guide and prioritize our analyses, aided in identification of data missing from our ecosystem, highlighted life-altering events that may lead to fragmented connections to care, helped interpret results, and suggested future interventions. This partnership has been effective in meeting each of the aims of our Getting to Zero project, with stakeholders having made a substantial impact on each.

### Aim 1: Analyze patterns in social services utilization among PLWH to understand correlates of poor HIV outcomes.

Stakeholder input informed the methods we used in our research on the impact of Medicaid enrollment discontinuity on HIV outcomes [29]. We not only examined discontinuity by enrollment type, but were able to share information on antecedents to continuous enrollment, disenrollment, or churn (disenrollment and subsequent re-enrollment within a brief period) based on their firsthand experience. This information is important to clinicians and case managers who became aware of difficulties faced by PLWH in the confusing and high-stakes process of applying and/or recertifying for this coverage. Stakeholder input also led us to integrate or explore social services data not previously considered by our team. Accessing CAREWare to evaluate the impact of Ryan White services is being considered as a method of evaluating the impact or interaction of Parts A/B/C coverage with other correlates of HIV health outcomes. We are also exploring unemployment and housing assistance data for inclusion in future analyses and have entered into an agreement to obtain Indiana eviction data.

### Aim 2a: Determine if mobility or migration affects HIV care outcomes.

Based on lessons learned from stakeholders regarding mobility and migration, we expanded Medicaid data to statewide coverage and leveraged our access to eHARS data for the 10-county Ryan White Part A TGA. These changes increased our cohort size, geographic diversity, and the strength of our findings.

### Aim 2b: Identify demographic, socioeconomic, life course (e.g., pregnancy, incarceration), and contextual factors associated with HIV outcomes and migration.

The stakeholder panel introduced our team to the idea of forced mobility (e.g., eviction, unsafe home) and shared negative and positive neighborhood characteristics (Table 4) that should be prioritized when planning a move or assisting PLWH in overcoming barriers faced due to residential location or circumstances. As a result, we are exploring use of unemployment and housing assistance data, incorporating statewide eviction data, integrating neighborhood characteristics (e.g., crime, income, public transit) into our studies, and searching out new sources of data to identify causes of mobility and migration and their interaction with other correlates of HIV outcomes. Stakeholders suggested that we evaluate time living with HIV, time after receiving a behavioral health diagnosis, time before or after incarceration, and/or the timing of other life course factors in relation to a move and/or HIV outcomes of interest. For this reason, our team will include time elements for future mobility analyses, including time since diagnosis, time spent retained in care prior to a move (proxy for system navigation experience), and date of last HIV health care (proxy for last provider contact).

### Aim 3: Extend the utility of eHARS for longitudinal research and public health practice.

Working with stakeholders, we learned that whether an individual was receiving regular HIV care was the most important consideration for generating an alert to case managers. Public health use of eHARS was considered the most straightforward for alerts because it is a complete record of HIV labs and because MCPHD’s RWHSP already employs staff to conduct outreach to PLWH who have fallen out of care. We learned that individuals are often identified for outreach several months after attrition from care because of the retrospective method being used to report retention in care. Also, outreach staff sometimes conducts outreach to healthy, virally suppressed individuals because viral load is not included in current methods of identification of attrition from care. We have engaged in an implementation project to leverage eHARS data, on MCPHD’s server, to proactively generate alerts to appropriately identify PLWH in need of outreach 30–60 days before they fall out of care. Once our program is implemented, we will evaluate and publish outcomes and will make the program freely available to other state and local health departments. Future work will include other ideas gleaned from the panel, such as working with the Indiana Health Information Exchange or with Indiana’s Family and Social Services Administration to generate alerts to case managers based on those data sources.

Perhaps the most profound way in which our research team and projects were impacted was by the shared trust and rapport with stakeholders, adding faces, firsthand experiences, and context to the data behind our work. This changed team attitudes toward the value of stakeholder engagement based on the experiences and relationships formed. Stakeholders shared that they also benefitted from participation. Jams enabled easy flow of information between clinicians, agency representatives, and PLWH. Multiple stakeholders left with pictures of Jam products and asked us to share meeting notes so they could use the information for their own agency-related work. Stakeholders living with HIV shared that playing a role on the panel served as a means of social support. One person emotionally shared that, “It felt good to be able to be our authentic selves,” and went on to share the difference he felt as he moved from feeling alone in his diagnosis to participating with others to improve care for PLWH.

Our use of the stakeholder panel was not without limitations and challenges. We were initially funded for this work before the COVID-19 pandemic, the onset of which notably shifted our approach. We did not meet stakeholders face-to-face until the fall of 2022. Related, at least in part, was an initial hesitancy of some stakeholders, particularly those living with HIV and/or from historically stigmatized backgrounds, to trust RJ or the research team enough to begin an open conversation about their personal experiences. Our interviews with stakeholders did, in fact, confirm their preference to meet in person and our initial inability to do so reduced our ability to build trusted relationships with stakeholders and slowed the flow of information. Also, while RJ made session insights available to stakeholders online via a Microsoft Teams platform with opportunities for between-session discussion and discovery, the stakeholder panel did not use this platform. When asked, stakeholders cited: (1) being too busy to participate off-session; (2) difficulty with technology; and (3) negative experiences with off-session communication in other groups of this type.

We worked with participants to identify other panel limitations. Stakeholders were concerned that the panel was missing individuals newly HIV diagnosed, and suggested placing someone with whom they could relate into a leadership role to increase recruitment and active participation. The local Ryan White Part A Planning Council conducts business in this manner, with experienced leaders mentoring younger up-and-coming leaders, and it works well for them. Stakeholders also expressed that the panel does not represent the diversity of experiences of PLWH. For example, SDOH and cultural differences were cited as a factor in one’s willingness to ask for assistance.

Translating findings to practice presents ethical and legal complexities. Expanded data access leads to new opportunities to discover and refine beneficial interventions [32]. For example, our work will alert public health practitioners to attrition from care *before* it happens, but social services data (e.g., eviction, Medicaid enrollment) could, in the future, be integrated into these alerts in order to make case managers aware of the need for social services navigation and/or wraparound services that can maintain good health among their clients. That being said, public health decisions based on insights identified through integration of ‘big data’ must balance the opportunity to intercede with obligations to minimize potential harms to the autonomy, dignity, trust, privacy, and confidentiality of affected individuals [33]. The stakeholders’ considerations and engagement were of great importance in this area. Our discussions on the balance of privacy and health led to the Reaching Out Decision Tree (Fig. 5).

## CONCLUSIONS

Community engagement expanded the scope of our research and provided value to both stakeholders and research team members. HCD enhanced this partnership by keeping it person-centered, developing empathy and trust, increasing stakeholder retention, and empowering stakeholders to externalize ideas and collaborate meaningfully with the research team. We recommend that stakeholder and community engagement, particularly with HCD methods, is essential to conducting relevant, impactful, and sustainable projects outside the realm of clinically-focused research. It can play a key role in any research that uses existing social, programmatic, and clinical data. We encourage readers to contact RJ (https://researchjam.org/) for information regarding the HCD tools described in this manuscript. We anticipate that these methods will be important for academic and public health researchers wishing to engage with, and integrate the ideas of, community stakeholders.

## Figures and Tables

**Table 1:** Getting to Zero Community Engagement Panel Demographics (N = 48)

	Female		Male		Non-Binary or Undisclosed		Total by Race/Ethnicity	
Race/Ethnicity	N	%	N	%	N	%	N	%
Non-Hispanic Black	7	14.6%	9	18.8%	0	0.0%	16	33.3%
Non-Hispanic White	11	22.9%	12	25.0%	0	0.0%	23	47.9%
Non-Hispanic Other	1	2.1%	0	0.0%	1	2.1%	2	4.2%
Hispanic/Latine	1	2.1%	3	6.3%	0	0.0%	4	8.3%
Undisclosed	1	2.1%	1	2.1%	1	2.1%	3	6.3%
**Total by Gender**	21	43.8%	25	52.1%	2	4.2%	48	100.0%

**Table 2:** Getting to Zero Jam Session Summaries, Marion County, Indiana: 2020–2023

Jam	Date (M/Y)	Virtual(Y/N)	Participants(N)	Composition	Focus Area	Activities
1	Oct-20	Y	10	PLWH, 2	Meeting; building rapport; investigating stakeholder needs and expectations	Hopes, Fears, and Ideals
				Clinicians, 2		
				Agency, 6		
2	May-21	Y	10	PLWH, 3	Risk and protective factors of a residential move; harmful and advantageous neighborhood characteristics	Collage; Personas (interpreting and creating); Mind maps;
				Clinicians, 3		
				Agency, 4		
3	Oct-21	Y	5	PLWH, 3	Data events useful to prompt alerts useful for case managers to check in with their clients living with HIV	Red Flag Game; Card Sorting Game; Decision Tree
				Clinicians, 1		
				Agency, 1		
4	May-22	Y	12	Inactive stakeholders	Ideas to increase jam session engagement	Interviews
5	Nov-22	N	17	PLWH, 6	HIV care engagement; residential mobility; Medicaid; arrest; comorbid mental health diagnosis; spatial relationships	Information Sharing Stations
				Clinicians, 1		
				Agency, 9		
6	Apr-23	N	12	PLWH, 6	Needs and available resources in the categories: Medical, Mental Health, Transportation, Housing, Social, Financial, and Other	Brainstorming; Resource Mapping
				Clinicians, 1		
				Agency, 5		

**Table 3:** Hopes, Fears, and Ideals of the Getting to Zero Community Engagement Panel

Hopes	Fears	Ideals
*Collaboration*	*Delays interfering with progress*	*Ways of working*
Shared space, ideas,	COVID and time between	Thinking outside the box
expertise	meetings	Better collaboration
*Increased resources*	*Who makes up the panel*	*Panel outcomes*
More funding	Lack of diversity	Reduce stigma
Personal development	‘Same player’ burn-out	Influence policy
Capitalize on increased	Not comfortable sharing thoughts	Increase services
awareness due to COVID	Bias affecting the panel	Retain PLWH in care
*Panel outcomes*	*Panel outcomes*	Improve patient
Use time wisely	Results not disseminated quickly	communication
Meaningful participation	No new solutions emerge	Connect agencies with
Meaningful outcomes	Panel not heard by researchers Known barriers unchanged	resources (i.e., funding, guidance with vulnerable populations)

**Table 4.** Harmful and Advantageous Neighborhood Characteristics Identified by the Getting to Zero Community Engagement Panel

Harmful Characteristics	Advantageous Characteristics
High crime, drug use, domestic violence	Care centers/clinics
High number of immigrants	Church pantries
High prevalence of mental health issues	Churches
Lacking convenient access to healthcare	HIV care
Lacking convenient access to nutritious foods	Mental health treatment centers
Lacking reliable public transportation	Pantries
Low access to childcare	Progressive policies (ex. Reproductive health care)
Low car and/or phone ownership	Public transportation
Low income/High unemployment	Public transportation for healthcare needs
Low or no use of available services	Ryan White supportive services
Low provider follow-up	Shopping centers
Neighborhoods that are declining	Small malls
Neighborhoods with high evictions/mobility	
Rural areas	

## Data Availability

We encourage readers to contact Indiana Clinical and Translation Sciences’ Research Jam (https://researchjam.org/) for additional information about the human-centered design tools described in this manuscript. Data generated and/or analyzed using these tools throughout the course of this project are available from the corresponding author upon reasonable request.

## Abbreviations

eHARS: Enhanced HIV/AIDS Reporting System
HCD: Human-centered design
HIV: Human immunodeficiency virus
MCPHD: Marion County Public Health Department
PLWH: People living with HIV
RJ: Research Jam
RWHSP: Ryan White HIV Services Program
SDOH: Social determinants of health
TGA: Transitional Grant Area
USPSTF: United States Preventive Services Task Force
